# The activation of glial cells in the medulla visceral zone impairs synaptic plasticity and promotes inflammation and immune disorders in sepsis

**DOI:** 10.1515/tnsci-2025-0398

**Published:** 2026-07-27

**Authors:** Jianjian Wang, Lizhi Wang, Weiwei Jiang, Lu Zhu, Hongbing Li

**Affiliations:** Thoracic Surgery Department of Hubei Cancer Hospital, Tongji Medical College, Huazhong University of Science and Technology, Wuhan, China; Infectious Diseases Department of Ezhou Central Hospital of Hubei Province, Ezhou, China; Emergency Department of the First People’s Hospital of Guiyang, Guiyang, China; General Practice Department of the First People’s Hospital of Guiyang, Guiyang, China

**Keywords:** glial cells, medulla visceral zone, synaptic plasticity, inflammation and immune, sepsis

## Abstract

**Objective:**

Sepsis elicits neuroinflammation in the medullary visceral zone (MVZ), which disrupts the cholinergic anti-inflammatory pathway (CAP). However, the underlying mechanisms and translational therapeutic potential remain poorly defined.

**Methods:**

Adult male Sprague–Dawley rats received either intracerebroventricular lipopolysaccharide (LPS; 25 μg/25 μL) to induce MVZ neuroinflammation model rats, or intraperitoneal LPS (6 mg/kg) to establish polymicrobial sepsis model rats. Anti-inflammation groups were involved with minocycline sucrose solution (120 mg/L) which was administered by drinking for 4 days prior to LPS challenge. CAP transection groups were undergone right cervical vagotomy 7 days before anti-inflammation interventions. Murine sepsis score (MSS), mortality rates, heart rate variability (HRV), cytokine levels in blood and medulla, systemic immunity level, and the expression levels of glial fibrillary acidic protein (GFAP), ionized calcium-binding adapter molecule 1 (IBA-1), CD22, tumor necrosis factor-α (TNF-α), Munc13-1, Synaptophysin (SYN), and postsynaptic density protein 95 (PSD-95) in the MVZ were measured and analyzed.

**Results:**

Both sepsis and MVZ neuroinflammation model rats showed significantly increased mortality and MSS scores, depressed HRV parameters, and upregulated expressions of GFAP, IBA-1, CD22, and TNF-α, with downregulated expressions of Munc13-1, SYN, and PSD-95 in the MVZ. Synaptic structural damage including reduced synaptic vesicles, decreased RT1.D expression on lymphocytes, increased percentages of Th17 and Treg lymphocytes, and increased serum levels of TNF-α, IL-10, and INF-γ were observed. Anti-inflammatory intervention reversed these changes in both model rats, whereas CAP transection partially abolished the anti-inflammatory benefits, confirming CAP dependence.

**Conclusions:**

Sepsis-induced MVZ neuroinflammation dismantles synaptic integrity and CAP modulation, which intensifies peripheral cytokine storm. Early central anti-inflammation repairs MVZ synaptic plasticity and reinstates vagal immunomodulation, which may provide a translational strategy to limit septic escalation.

## Introduction

Previous studies have confirmed that [1], 2] sepsis-induced neuroinflammation in the medullary visceral zone (MVZ) leads to functional inhibition of the cholinergic anti-inflammatory pathway (CAP), which plays a key role in the early inflammatory storm of sepsis. The time and frequency domain indexes of heart rate variability (HRV) can reflect the regulatory effect of the CAP and the degree of MVZ inflammation. Intervention in MVZ neuroinflammation can effectively curb the inflammatory storm of sepsis. Furthermore, the severity and prognosis of sepsis can be evaluated by monitoring HRV indexes, which provides a new potential target for neurological intervention and treatment efficacy evaluation for sepsis.

It is well established that glial cells play a decisive role in the occurrence and maintenance of neuroinflammation. Inflammation, infection, or nerve injury in the central nervous system (CNS) can activate glial cells, thereby regulating the immune response and inflammation within the CNS [3]. Microglia are the most important immune cells in the CNS with a high degree of plasticity. They can change different phenotypes and functions in response to specific microenvironmental signals. Microglia are key regulators of synaptic pruning and remodeling; they can cause synaptic loss [4], nerve injury, and neuronal loss [5] under inflammatory conditions. Reactive astrocytes activated by inflammation promote the imbalance of oxidation-reduction reactions by releasing cytokines, inflammatory mediators, nitric oxide, and reactive oxygen species, which also lead to nerve injury [6]. In addition, reactive astrocytes can induce the reprogramming of microglia to convert into ionized calcium binding adapter molecule 1 (Iba-1) labeled pro-inflammatory microglia [7], which further aggravates neuroinflammation and nerve injury. Whether sepsis aggravates MVZ neuroinflammation and interferes with CAP modulation of systemic inflammation by activating MVZ glial cells remains unclear. Moreover, the selective microglia inhibitor minocycline has anti-neuroinflammatory, immunomodulatory, and neuroprotective effects [8]. Whether minocycline can dampen MVZ neuroinflammation and improve the modulation of CAP on systemic inflammation through its central anti-inflammatory role needs further study.

The structural integrity and functional normality of synapses are prerequisites for normal nervous information conduction. Synaptic dysfunction causes neural network disorders, which are closely related to almost all CNS diseases, such as epileptic seizures in patients with Alzheimer’s disease [9] and cognitive impairment induced by chronic cerebral hypoperfusion [10]. Therefore, sepsis-induced MVZ neuroinflammation likely leads to synaptic dysfunction, contributing to the dysregulation of CAP on systemic inflammation and immunity. The specific mechanisms still need to be explored. Munc13-1, a mammalian homolog of *C. elegans* unc-13p, is a key protein required for synaptic vesicle fusion and neurotransmitter release in the brain [11]. Munc13-1 is essential for vesicle docking and fusion in the synaptic active zone and is also a key protein in the regulation of synaptic plasticity [12]. Among the postsynaptic components, the postsynaptic density-95 protein (PSD-95) is closely related to synaptic maturation and synaptic plasticity [13], 14]. Presynaptic Munc13-1 and postsynaptic PSD-95 form well-aligned nanopillar arrays that precisely regulate synaptic vesicle release [15]. Synaptophysin (SYN) is a 38 kDa presynaptic vesicle-specific protein composed of 313 amino acids, found in all neurons and can be used as a marker of synaptic content [16]. SYN and PSD-95 are markers of synaptic structure and key proteins that mediate long-term potentiation (LTP) [16]. The expression of these proteins in the MVZ in sepsis and their effects on CAP modulation, systemic inflammation, and immunity need to be further explored to provide insights for neurological intervention in sepsis.

## Materials and methods

### Experimental animals

A total of 112 specific pathogen-free (SPF) male Sprague-Dawley (SD) rats (180–220 g) were purchased from the Hubei Provincial Laboratory Animal Research Center (license No. SCXK (Hubei) 2020-0018). Animals were housed at Guizhou Medical University, with ad libitum access to food and water. The environmental conditions were maintained on a 12-h light/dark cycle, at a temperature of 22 ± 2 °C, and a humidity of 60 ± 10 %. After a 7-day acclimatization period, the experiments were initiated. All procedures complied with the Guide for the Care and Use of Laboratory Animals (IACUC) and were approved by the Animal Ethics Committee of Guizhou Medical University (approval No. 2100819).

### Animals grouping and treating

**Figure 1:**
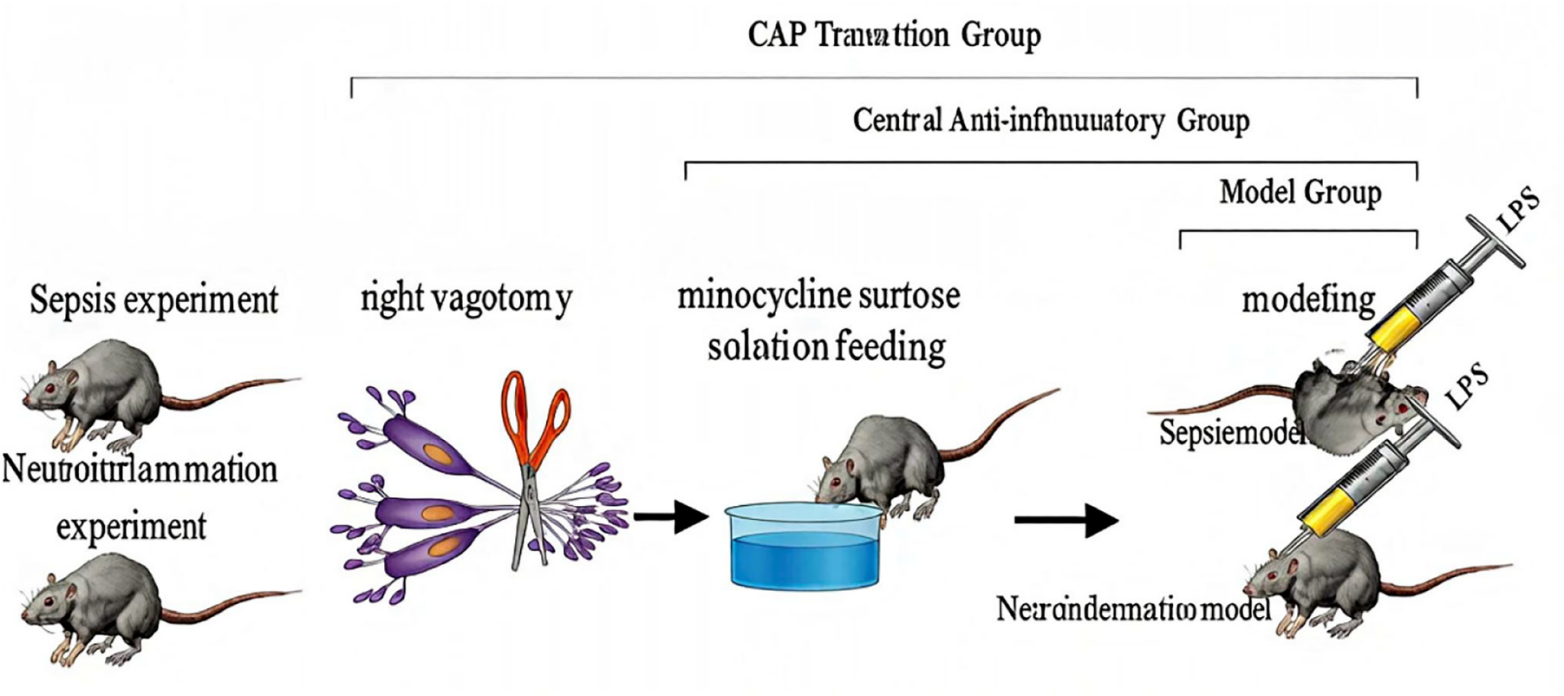
Rats grouping and treatment.

The rats were randomly assigned to either the sepsis experimental group or the MVZ neuroinflammation experimental group. Each group was further divided into four subgroups: Control Group (n=8), Model Group (n=16), Anti-inflammatory Group (n=16), and CAP Transection Group (n=16). The intervention methods for each group are detailed as follows (refer to Figure 1):

① Control Group: Rats in the sepsis Control Group received intraperitoneal injection of 6 mg/kg normal saline. Rats in the MVZ neuroinflammation Control Group were administered 25 μL of artificial cerebrospinal fluid (ACSF), which was injected into the fourth ventricle. The composition of ACSF was as follows [17]: sodium chloride 6.279 g, potassium chloride 0.216 g, calcium chloride 0.353 g, magnesium chloride 0.488 g, sodium bicarbonate 1.932 g, glucose 0.6 g, disodium hydrogen phosphate 0.358 g, with purified water added to a total volume of 1000 mL.
② Model Group: Rats in the sepsis Model Group were injected intraperitoneally with lipopolysaccharide (LPS) at a dosage of 6 mg/kg. Rats in the MVZ neuroinflammation Model Group received 25 μg LPS dissolved in 25 μL ACSF, which was injected into the fourth ventricle.
③ Anti-inflammatory Group: Rats in both Anti-inflammatory Groups were provided with 10 mg/mL minocycline phosphate (dissolved in a 5 % sucrose solution) as their sole source of drinking water for four days prior to the induction of sepsis or MVZ neuroinflammation models [18]. The rats were maintained on minocycline sucrose solution throughout the experiment. The rats had an average daily water intake of about10 mL, the daily minocycline dose per rat was 10 mg/mL × 10 mL=100 mg, with a total continuous treatment duration of 7 days.
④ CAP Transection Group: Following the separation and transection of the right cervical vagus nerve, rats in both CAP Transection Groups were allowed a 7-day recovery period before receiving treatments identical to their respective central Anti-inflammatory Groups.

For fourth ventricle localization and injection, the anterior fontanel was used as a reference point, with the fourth ventricle’s body surface projection located approximately 11.6 mm posterior to the central point of the anterior fontanel along the midline. Under stereotaxic guidance, rats were secured after anesthesia, and a small incision was made at the body surface projection of the fourth ventricle. A trace injector needle was inserted vertically to a depth of 7.5–8.5 mm into the fourth ventricle. Once cerebrospinal fluid could be aspirated, solutions such as LPS or normal saline were slowly injected. The needle was left in place for 5 min to ensure complete absorption before being withdrawn.

In accordance with IACUC guidelines, rats were anesthetized using isoflurane inhalation for both fourth ventricle injections and sacrifices for sampling.

### Murine sepsis score (MSS) and rats’ mortality rates

The MSS scoring system was employed to evaluate the severity of disease, is reasonably confirmed that successful septic models established when the MSS of rat exceeded to 4 points, the higher the MSS, the serious the model rat [19]. Three experimental technicians independently scored all rats, and the average score was taken as the final score for each rat. Additionally, mortality rates within each group were recorded and analyzed.

### Evaluation of CAP’s modulation

Heart rate variability (HRV) was utilized to assess the modulation strength of the CAP, as per previous research [2]. A 5-min short-term electrocardiogram (ECG) was recorded to analyze HRV indices, including time domain measures such as the standard deviation of all RR intervals (SDNN) and the root mean square difference of successive RR intervals (RMSSD). For frequency domain measures, Lomb-Scargle cycle plots were applied to estimate the power spectral density and determine the low-frequency (LF, 0.2–0.75 Hz) and high-frequency (HF, 0.75–2.5 Hz) power components, calculating the LF/HF ratio. The ECG data were processed using the BL-420F biological signal acquisition and analysis system from Chengdu Techman Software to calculate time domain and frequency domain indices.

### Sampling blood and the medulla oblongata

Following the completion of the experiments, surviving rats from each group were anesthetized via isoflurane inhalation. Their chests were opened to expose the thoracic cavity, and blood samples (8 mL) were collected from the right ventricle. Samples were left to stand for 1 h, centrifuged at 3,000 rpm for 10 min, and the serum was harvested. After blood sampling, rats were transcardially perfused with phosphate-buffered saline (PBS, 0.01 M, pH 7.4) until their organs blanched, followed by perfusion with paraformaldehyde (PFA, 350 mL) solution. The medulla oblongata, including the dorsal vagus motor nucleus (DVMN), the nucleus of the solitary tract (NST), and the rostral ventrolateral medulla (RVLM), were then isolated and stored at −20 °C for subsequent analysis.

### ELISA detection and flow cytometry analysis

The ELISA procedure was conducted according to the kit’s instructions. Rats’ serum (10 μL) was added to the sample wells of the plate, with corresponding standard wells also set up, gently mixed, and incubated at 37 °C for 2 h. After emptying and drying the wells, 100 μL of antibody-labeled working solution was added, including Rat TNF-α, Rat IL-10, and Rat INF-γ (producer: Enzyme Immunoassay, with respective batch numbers MM-0180R1, MM-0195R1, MM-0198R1). Wells were incubated at 37 °C for 1 h, washed, and 50 μL of chromogen A and B were added. After a 10-min reaction, the experiment was stopped, and the optical density (OD) values of each well were measured at 450 nm using a microplate reader (producer: Multiskan FC, model: Thermo Scientific). Concentrations of TNF-α, IL-10, and INF-γ were calculated.

Rat-specific anti-RT1.D antibody (the functional homolog of human HLA-DR in rats, produced by invitrogen, Cat. No. MA5-32232) was used to detect MHC class II molecule expression on rat lymphocytes. IL-17 fluorescent antibody was used to mark TH17 lymphocytes, and Foxp3+fluorescent antibody was used to mark Treg lymphocytes. The percentages of Th17 and Foxp3+Treg cells and the ratio of Foxp3+Treg/Th17 were detected by flow cytometry.

### Immunohistochemical staining

Brain tissue blocks from the middle and caudal segments of the medulla oblongata (from the area postrema to the obex) were collected, embedded in paraffin, and sectioned into 30 μm slices. After deparaffinization and rehydration, tissue sections were subjected to antigen retrieval by boiling in 0.01 M citrate buffer (pH 6.0) for 15 min and cooling to room temperature naturally. Sections were blocked with 5 % bovine serum albumin (BSA) in 0.01 M PBS (pH 7.4) containing 0.3 % Triton X-100 for 1 h at room temperature to block non-specific binding. Primary antibodies (anti-GFAP, ab68428, 1:250; anti-Iba-1, ab178846, 1:200; anti-CD11b, ab133357, 1:200) were added and incubated at 4 °C for 15 h. After washing 3 times with PBS (5 min each), sections were incubated with horseradish peroxidase (HRP)-conjugated goat anti-rabbit secondary antibody (1:500) for 1 h at room temperature. After another 3 washes with PBS, freshly prepared DAB chromogenic solution was added for 3–5 min (monitored under a light microscope to avoid over-coloration). The reaction was terminated with distilled water, followed by hematoxylin counterstaining for 1 min, differentiation with 1 % hydrochloric acid alcohol for 30 s, and bluing with 0.5 % ammonia water. The total number of cells and the number of positive cells in each field were counted at 400 × magnification. Two different areas per section and six sections per group were analyzed, and the average ratio of positive cells to total cells was calculated among different groups.

### Western blot analysis

A small amount of medulla oblongata tissue from each group was placed into 2 mL EP tubes, and 200 μL of RIPA lysate (producer: Meilunbio, batch number: MA0151) was added for cell lysis. Total protein was extracted by centrifugation, and protein concentration was determined using the BCA method. Equal amounts of protein were loaded, denatured, and electrophoresed on polyacrylamide gels, then transferred to polyvinylidene difluoride (PVDF) membranes. Membranes were blocked with 8 % skim milk powder at room temperature for 1 h, followed by incubation with primary antibodies, including rabbit polyclonal antibody TNF-α (21KD) (producer: Biyuntian, batch number: AF8208), rabbit polyclonal antibody of post-synaptic density (PSD)-95 (105KD) (producer: Affinity, batch number: AF5283), rabbit polyclonal antibody of Munc13-1 (200KD) (producer: Abcam, batch number: Ab307511), and rabbit polyclonal antibody of synaptophysin (SYP) (38KD) (producer: Affinity, batch number: AF0257), overnight at 4 °C on a shaker. The next day, membranes were washed and incubated with horseradish peroxidase (HRP)-labeled sheep anti-rabbit secondary antibody (producer: Wuhan Bade Biological Engineering Co., LTD., batch number: BA1054) for 2 h at room temperature on a shaker. Electrochemiluminescence (ECL) affinity (producer: Affinity, batch number: KF8003) was used to image and expose the films on a gel imager (producer: Hangzhou Shenhua Technology Co., LTD., batch number: SH-523). The gray value of the films was analyzed using ipp6.0 software to determine protein expression levels.

### Statistics

All measurement data are expressed as mean ± standard deviation ( $\overset{®}{X}$ ± S), and statistical analysis was performed using SPSS 22.0 software. Measurement data were analyzed using one-way analysis of variance (ANOVA), with the Levene test for homogeneity of variances. For homogeneous variances, the Bonferroni test was applied; otherwise, Tamhane’s test was used. Count data were analyzed by the Chi-square (χ^2^) test. p<0.05 was considered statistically significant.

## Results

### The mortality rate and MSS among different groups

In the sepsis experiment, the mortality rates for the Control Group, Model Group, Anti-inflammatory Group, and CAP Transection Group were 0 %, 36.84 %, 21.43 %, and 31.25 %, respectively. The survival rate of the Model Group was significantly lower than that of the Control Group (χ^2^=4.563, p=0.033). In the neuroinflammation experiment, the mortality rates for the Control Group, Model Group, Anti-inflammatory Group, and CAP Transection Group were 0 %, 52.63 %, 28.57 %, and 43.75 %, respectively. The survival rates of the Model Group and the CAP Transection Group were significantly lower than those of their respective Control Groups (χ^2^=7.829, p=0.005; χ^2^=4.735, p=0.030). Central anti-inflammation with minocycline significantly improved the survival rate of the neuroinflammation model (χ^2^=4.843, p=0.028).

Both sepsis and MVZ neuroinflammation led to a significant increase in MSS scores in rats. Central anti-inflammation with minocycline significantly reduced MSS scores in the sepsis and neuroinflammation Model Groups, while right vagotomy abolished the reduction of MSS scores caused by central anti-inflammation, resulting in a significant increase in MSS scores (Table 1).

**Table 1:** The MSS among different groups.

	Sepsis experiment		Neuroinflammation experiment	
	n	MSS	n	MSS
Control Group	8	0	8	0
Model Group	16	24.29 ± 2.21^a^	16	26.43 ± 1.99^a^
Central anti-inflammation group	16	5.43 ± 0.98^ab^	16	14.29 ± 2.87^ab^
CAP rransection group	16	17.29 ± 0.95^abc^	16	18.71 ± 2.69^abd^

^a^p<0.001 vs. Control Group; ^b^p<0.001 vs. Model Group; ^c^p<0.001 vs. central anti-inflammation group; ^d^p<0.01 vs. central anti-inflammation group.

### The expressions of GFAP, Iba-1 and CD11b in MVZ

Immunohistochemical analysis indicated that in both sepsis and neuroinflammation experiments, there was a significant up-regulation in the expressions of GFAP, Iba-1, and CD11b in the Model Group when compared to the Control Group. These expressions were significantly reduced in the Anti-inflammatory Group compared to the Model Group. However, in the CAP Transection Group, the central anti-inflammatory effect was significantly diminished, leading to a notable increase in the expressions of GFAP, Iba-1, and Panel a, b and c are representative immunohistochemical expression images and their local magnification images of GFAP, Iba-1 and CD11b in each group. These images show that the expressions of GFAP, Iba-1 and CD11b were significantly increased in both sepsis and MVZ neuroinflammation models compared to their individual control groups. The central anti-inflammatory effect with minocycline significantly reduced the expression of these proteins, while right vagotomy partly abolished the central anti-inflammatory effect. Plots d, e and f show the percentage of GFAP, Iba-1 and CD11b positive cells to the total number of cells (positive percentage). In the sepsis experiment, there was no significant difference in the total number of cells in each group (p>0.05), but the positive percentage of GFAP, Iba-1 and CD11b in the Model Group were significantly higher than those in the Control Group (29.02 % ± 4.29 % vs. 7.52 % ± 3.50 %, p=0.000; 17.70 % ± 2.70 % vs. 6.89 % ± 1.40 %, p=0.000; 7.98 % ± 1.55 % vs. 1.37 % ± 0.60 %, p=0.000). Central anti-inflammation significantly reduced the positive percentage of GFAP, Iba-1 and CD11b in sepsis (17.56 % ± 2.02 % vs. 29.02 % ± 4.29 %, p=0.000; 10.25 % ± 1.49 % vs. 17.70 % ± 2.70 %, p=0.000; 3.56 % ± 1.04 % vs. 7.98 % ± 1.55 %, p=0.000), while right vagotomy almost abolished the central anti-inflammatory effect, increase the positive percentage of GFAP, Iba-1 and CD11b (23.36 % ± 4.78 % vs. 17.56 % ± 2.02 %, p=0.001; 14.48 % ± 3.41 % vs. 10.25 % ± 1.49 %, p=0.000; 6.31 % ± 1.47 % vs. 3.56 % ± 1.04 %, p=0.000). Similarly, in the neuroinflammation experiment, there was no difference in the total number of cells in each group (p>0.05), but the positive percentage of GFAP, Iba-1 and CD11b in the neuroinflammation model group were significantly higher than those in the Control Group (16.80 % ± 4.00 % vs. 6.77 % ± 1.92 %, p=0.000; 15.14 % ± 2.63 % vs. 6.77 % ± 2.54 %, p=0.000; 7.26 % ± 2.03 % vs. 2.26 % ± 1.01 %, p=0.000). Central anti-inflammation by minocycline significantly reduced the positive percentage of GFAP, Iba-1 and CD11b in the neuroinflammation model rats (9.56 % ± 3.10 % vs. 16.80 % ± 4.00 %, p=0.000; 9.64 % ± 2.24 % vs. 15.14 % ± 2.63 %, p=0.000; 3.57 % ± 0.84 % vs. 7.26 % ± 2.03 %, p=0.000), while right vagotomy evidently abolished the central anti-inflammatory effect, promote positive percentage of GFAP, Iba-1 and CD11b (13.46 % ± 3.18 % vs. 9.56 % ± 3.10 %, p=0.000; 11.64 % ± 1.73 % vs. 9.64 % ± 2.24 %, p=0.040; 5.93 % ± 1.52 % vs. 3.57 % ± 0.84 %, p=0.004).

Scale bar: 50 μm.

Note: ***p<0.001; **p<0.01; *p<0.05.

### Expressions of TNF-a and synaptic plasticity associated proteins in MVZ

**Figure 2:**
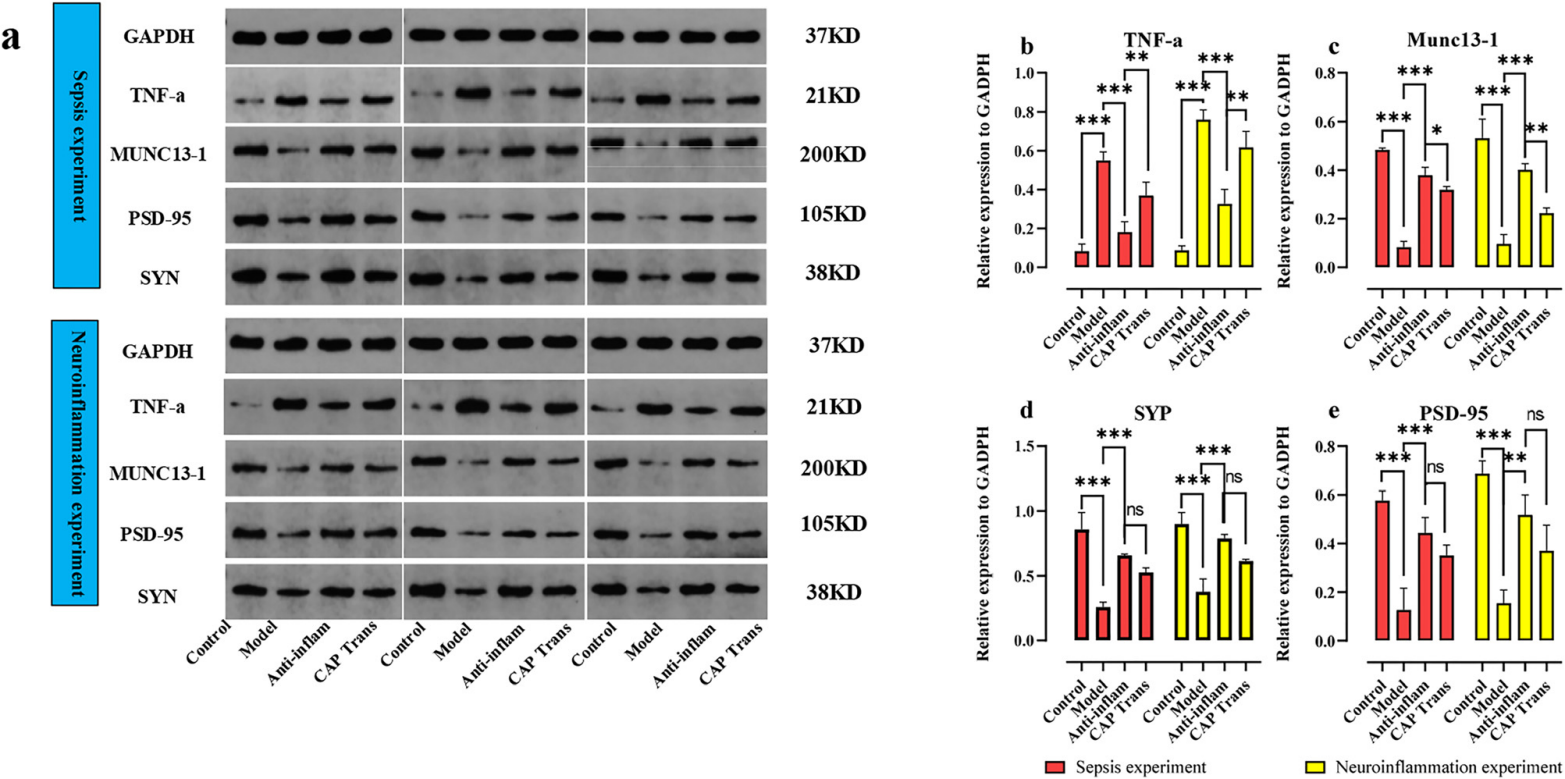
Expression of TNF-a and synapse plasticity associated proteins in the MVZ.

In the sepsis and neuroinflammation experiments, the expression levels of TNF-α in the MVZ were significantly elevated in the Model Group compared to the Control Group. Concurrently, the expressions of Munc13-1, synaptophysin (SYP), and postsynaptic density protein 95 (PSD-95) were significantly reduced in the Model Group relative to the Control Group. Central anti-inflammatory treatment effectively mitigated these changes by significantly reducing TNF-α expression and increasing the expressions of Munc13-1, SYP, and PSD-95. However, CAP transection partially reversed the effects of central anti-inflammatory treatment, leading to a significant upregulation of TNF-α and downregulation of Munc13-1, SYP, and PSD-95 (Figure 2). Panel a shows the western blotting bands images of the expressions of TNF-a, Munc13-1, SYP and PSD-95 in the MVZ. Panels b, c, d, and e show the relative expression of TNF-a, Munc13-1, SYP, and PSD-95 to the expression of GAPDH in each group. It can be seen that the expression of TNF-a in the sepsis Mosel Group was significantly higher than that in the Control Group (0.551 ± 0.044 vs. 0.083 ± 0.039, p=0.000). In the Model Group, the expressions of Munc13-1, SYN and PSD-95 were significantly down-regulated compared to those in the control group (0.083 ± 0.024 vs. 0.484 ± 0.007, p=0.000; 0.258 ± 0.039 vs. 0.858 ± 0.131, p=0.000; 0.127 ± 0.090 vs. 0.578 ± 0.039, p=0.000). Central anti-inflammatory treatment significantly reduced the expression of TNF-a in the sepsis Model Group (0.181 ± 0.054 vs. 0.551 ± 0.044, p=0.000), and also increased the expressions of Munc13-1, PSD-95 and SYP (0.379 ± 0.033 vs. 0.083 ± 0.024, p=0.000; 0.658 ± 0.009 vs. 0.258 ± 0.039, p=0.000; 0.444 ± 0.064 vs. 0.127 ± 0.090, p=0.000), while right vagotomy obviously abolished the effect of central anti-inflammatory and increased the expression of TNF-a (0.370 ± 0.069 vs. 0.181 ± 0.054, p=0.002). At the same time, the expression of Munc13-1, SYP and PSD-95 decreased, though the comparisons of SYP and PSD-95 didn’t show significant difference (0.320 ± 0.014 vs. 0.379 ± 0.033, p=0.011; 0.527 ± 0.034 vs. 0.658 ± 0.009, p=0.052; 0.351 ± 0.043 vs. 0.444 ± 0.064, p=0.105). Similarly, in the neuroinflammation experiment, the expression of TNF-a in the neuroinflammation Model Group was significantly higher than that in the Control Group (0.761 ± 0.051 vs. 0.087 ± 0.023, p=0.000). In the Model Group, the expressions of Munc13-1, SYP and PSD-95 were significantly lower than those of the Control Group (0.097 ± 0.039 vs. 0.533 ± 0.077, p=0.000; 0.377 ± 0.098 vs. 0.901 ± 0.088, p=0.000; 0.154 ± 0.055 vs. 0.687 ± 0.052, p=0.000). Central anti-inflammatory treatment significantly reduced the expression of TNF-a in the neuroinflammation Model Group (0.328 ± 0.074 vs. 0.761 ± 0.051, p=0.000), and also increased the expressions of Munc13-1, PSD-95 and SYP (0.402 ± 0.026 vs. 0.097 ± 0.039, p=0.000; 0.788 ± 0.032 vs. 0.377 ± 0.098, p=0.002; 0.519 ± 0.083 vs. 0.154 ± 0.055, p=0.000), while right vagotomy evidently abolished the central anti-inflammatory effect and increased the expression of TNF-a (0.618 ± 0.083 vs. 0.328 ± 0.074, p=0.003). Meanwhile, the protein expressions of Munc13-1, SYP and PSD-95 significantly decreased, the expressions of SYP and PSD-95 showed decreased trend (0.223 ± 0.022 vs. 0.402 ± 0.026, p=0.009; 0.613 ± 0.015 vs. 0.788 ± 0.032, p=0.083; 0.371 ± 0.105 vs. 0.519 ± 0.083, p=0.570).

Note: ***p<0.001;**p<0.01, *p<0.05; ns p>0.05.

### HRV study

**Figure 3:**
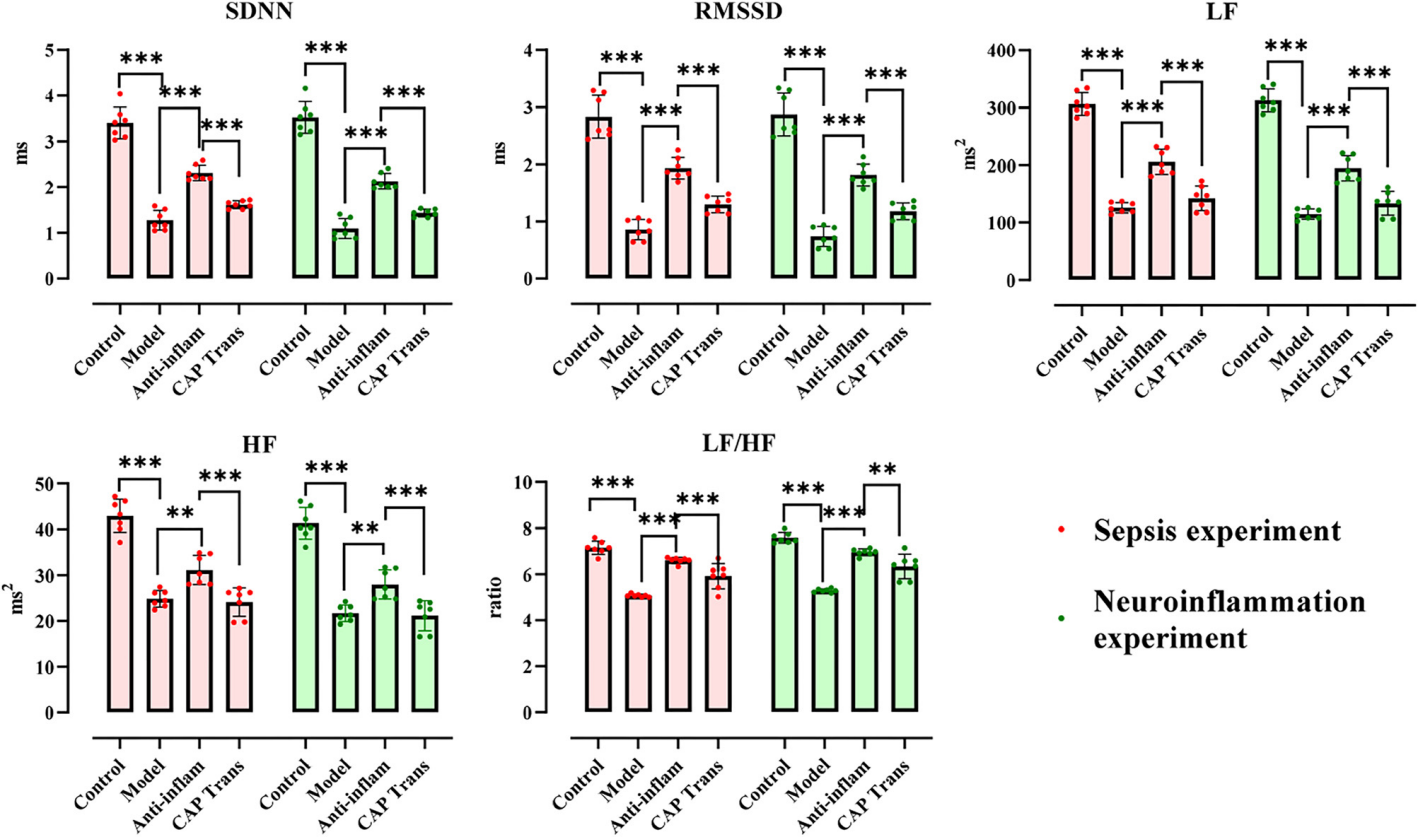
The indexes of HRV among different groups.

In both sepsis and neuroinflammation experiments, the heart rate variability (HRV) indices – specifically, the standard deviation of all RR intervals (SDNN), the root mean square difference of successive RR intervals (RMSSD), low-frequency (LF), high-frequency (HF) components, and the LF/HF ratio – were significantly reduced in the Model Group compared to the Control Group. Central anti-inflammatory treatment significantly reversed these HRV changes induced by the experimental models. However, the central anti-inflammatory effects were largely negated by CAP transection, as evidenced by a return towards the reduced HRV indices observed in the Model Group. Refer to Figure 3 for a detailed illustration of these findings, see Figure 3. HRV indexes such as SDNN (ms), RMSSD (ms), LF (ms^2^), HF (ms2), LF/HF in the sepsis model group were significantly lower than those in the control group, the comparison results were respectively 1.28 ± 0.22 vs. 3.40 ± 0.35, p=0.000; 0.86 ± 0.18 vs. 2.83 ± 0.37, p=0.000; 126.00 ± 9.17 vs. 306.45 ± 19.76, p=0.000; 24.85 ± 1.81 vs. 42.94 ± 3.61, p=0.000; 5.07 ± 0.07 vs. 7.15 ± 0.2, p=0.000; they turned significantly higher in the anti-inflammatory group than those in the model group, the comparison results of SDNN (ms), RMSSD (ms), LF (ms^2^), HF (ms2), LF/HF were respectively 2.31 ± 0.17 vs. 1.28 ± 0.22,p=0.000; 1.93 ± 0.19 vs. 0.86 ± 0.18, p=0.000; 205.94 ± 22.17 vs. 126.00 ± 9.17, p=0.000; 31.15 ± 3.19 vs. 24.85 ± 1.81, p=0.001; 6.61 ± 0.13 vs. 5.07 ± 0.07, p=0.000; they became much lower in the CAP transection group again than those in the anti-inflammatory group, the comparison results of SDNN (ms), RMSSD (ms), LF (ms^2^), HF (ms2), LF/HF were respectively 2.31 ± 0.17 vs. 1.28 ± 0.22, p=0.000; 1.93±0.19 vs. 0.86 ± 0.18, p=0.000; 205.94 ± 22.17 vs. 126.00 ± 9.17, p=0.000; 31.15 ± 3.19 vs. 24.85 ± 1.81, p=0.001; 6.61 ± 0.13 vs. 5.07 ± 0.07, p=0.000. There was no significant difference in LF and HF between the model group and the CAP transection group (p=0.116; p=0.464). Similarly, HRV parameters in the MVZ neuroinflammation model group were much lower than those in the control group, the comparison results of SDNN (ms), RMSSD (ms), LF (ms^2^), HF (ms2), LF/HF were respectively 1.10 ± 0.22 vs. 3.52±0.35, p=0.000; 0.74 ± 0.18 vs. 2.87 ± 0.37, p=0.000; 114.76±9.17 vs. 31 2.76 ± 19.76, p=0.000; 21.70 ± 1.81 vs. 41.32 ± 3.49, p=0.000; 5.29 ± 0.08 vs. 7.58 ± 0.23, p=0.000; HRV parameters in the anti-inflammatory group were significantly higher than those in the model group [SDNN (ms): 2.13 ± 0.17 vs. 1.10 ± 0.22, p=0.000; RMSSD (ms): 1.81 ± 0.19 vs. 0.74 ± 0.18, p=0.000; LF (ms2): 194.70 ± 22.17vs 114.76 ± 9.17, p=0.000; HF(ms2): 28.00 ± 3.19 vs. 21.70 ± 1.81, p=0.001; LF/HF: 6.95 ± 0.14 vs. 5.29 ± 0.08, p=0.000]; HRV parameters in the CAP transection group were obviously lower than those in the anti-inflammatory group [SDNN (ms): 1.44 ± 0.08 vs. 2.13 ± 0.17, p=0.000; RMSSD (ms): 1.18 ± 0.15 vs. 1.81 ± 0.19, p=0.000; LF (ms2): 133.68 ± 21.06 vs. 194.70 ± 22.17, p=0.000; HF (ms2): 21.16 ± 3.31 vs. 28.00 ± 3.19, p=0.000; LF/HF: 6.34 ± 0.53 vs. 5.29 ± 0.08, p=0.001]. There was no significant difference in LF and HF between the model group and the CAP transection group (p=0.071; p=0.741).

Note: ***p<0.001;**p<0.01.

### Systemic immunity and inflammation

**Figure 4:**
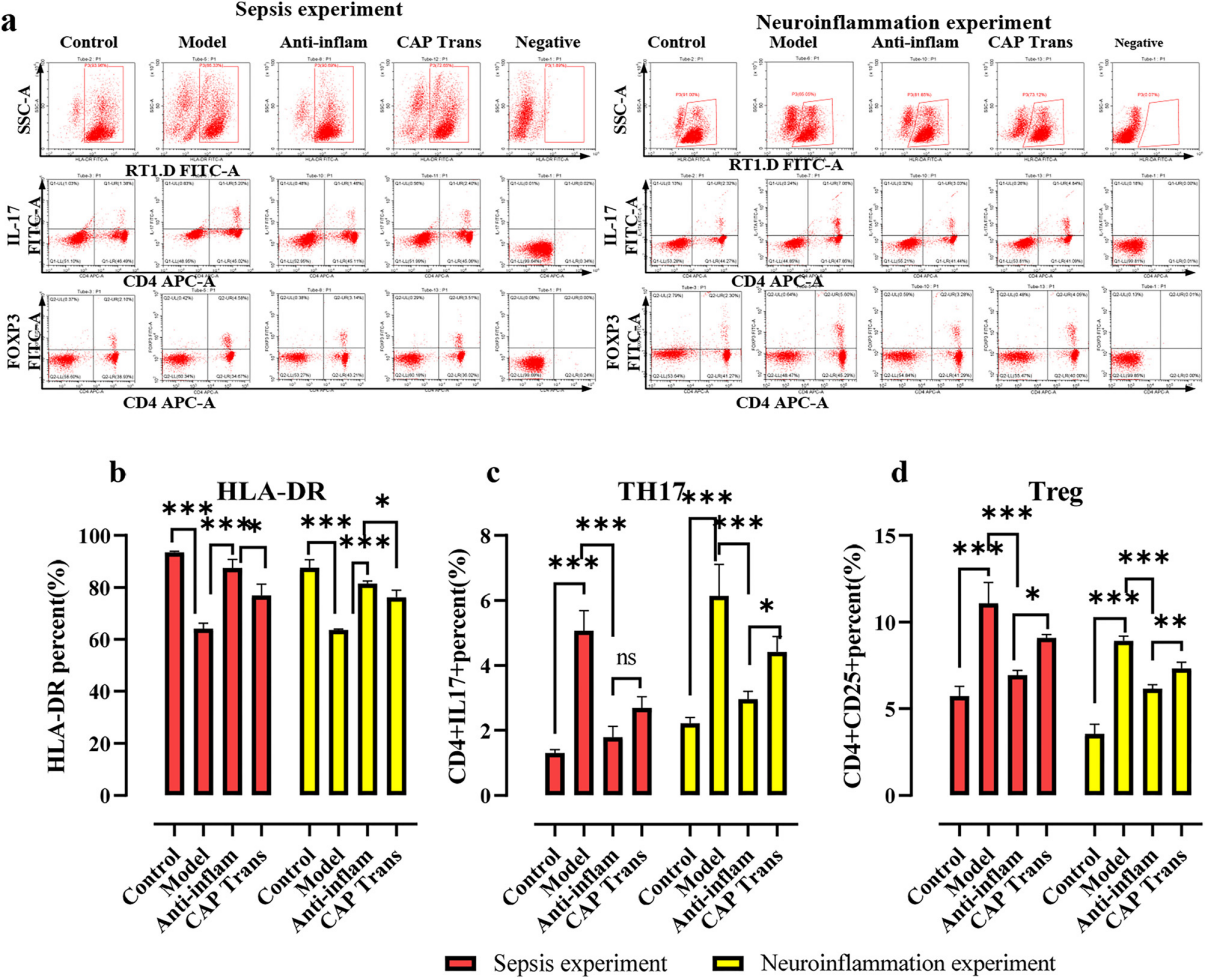
The expression percent of RT1.D+, CD4+IL17+, CD4+FOXP3+ among different groups. a: Representative images of flow cytometry among different groups. b–d: Show the histograms of expression percent of RT1.D, TH17 lymphocytes percent and treg lymphocyte percent among different groups.

In both the sepsis and neuroinflammation experiments, the expression of RT1.D in the Model Group was significantly downregulated compared to the Control Group, and there was a significant increase in the percentages of TH17 and Treg lymphocytes. Central anti-inflammatory treatment with minocycline significantly upregulated the expression of RT1.D and reduced the percentages of TH17 and Treg lymphocytes in the model rats. However, CAP transection partially reversed the central anti-inflammatory effects, significantly downregulating RT1.D expression in the Anti-inflammatory Groups of both experiments and significantly increasing the percentages of Treg lymphocytes in both experiments. In the neuroinflammation experiment, CAP transection also significantly increased the percentage of TH17 lymphocytes and showed a tendency to increase TH17 lymphocyte percentages in the sepsis experiment (Figure 4).

**Figure 5:**
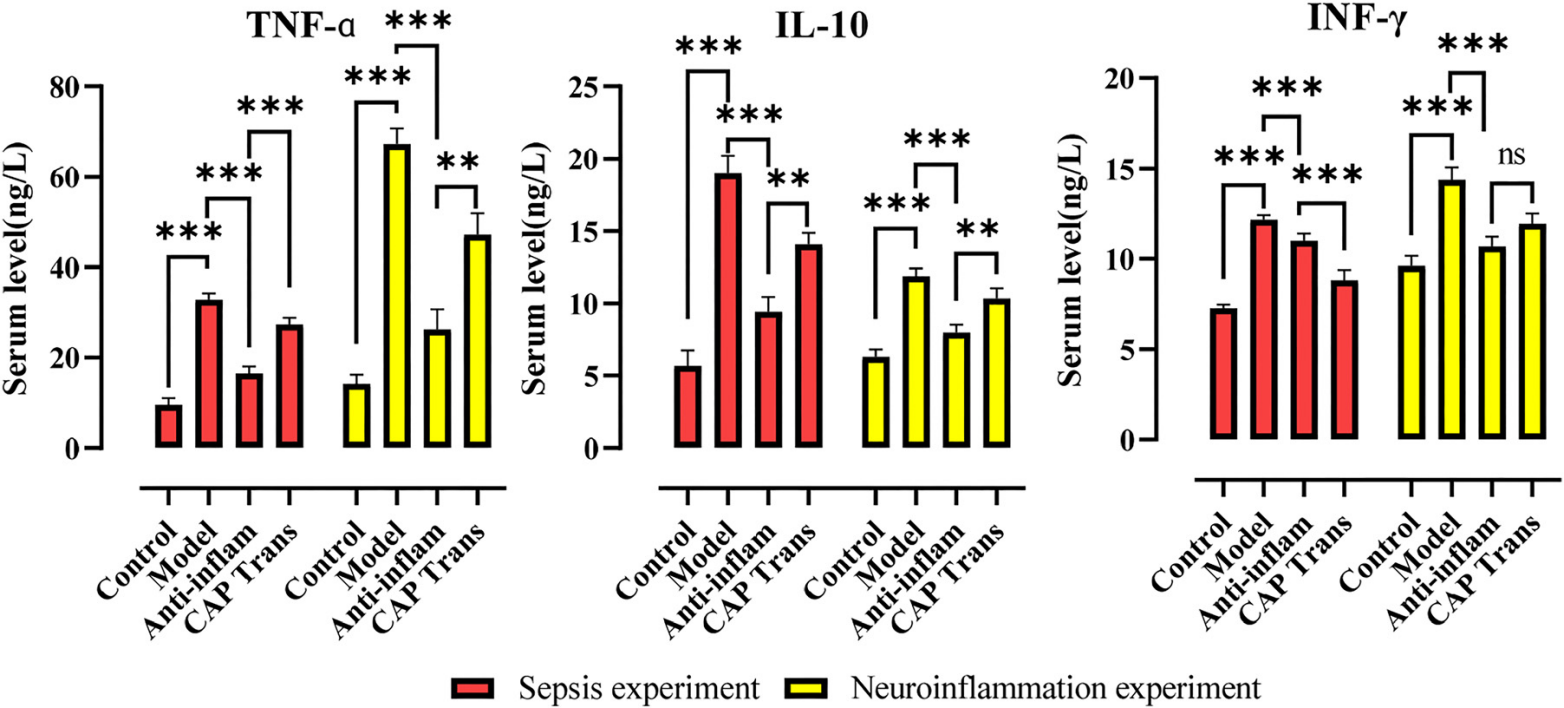
The serum concentration of TNF-a, IL-10, INF-γ among different groups.

Regarding systemic inflammation, both sepsis and neuroinflammation experiments demonstrated that the levels of TNF-α, IL-10, and INF-γ in the peripheral blood of the Model Group were significantly elevated compared to the Control Group. Central anti-inflammatory treatment with minocycline significantly reduced these inflammatory cytokine levels in the Model Group. However, CAP transection partially abrogated the central anti-inflammatory effects, leading to significant increases in TNF-α and IL-10 levels in both experiments and INF-γ levels in the sepsis experiment. In the neuroinflammation experiment, INF-γ levels showed a tendency to increase (Figure 5).

The results of flow cytometer suggests that the expression percent of RT1.D significantly decreased in the sepsis Model Group than that in the Control Group(64.18 % ± 2.05 % vs. 93.50 % ± 0.48 %, p=0.000), on the contrary, the percents of TH17 (CD4+IL17+) and Treg (CD4+FOXP3+) lymphocyte in the sepsis Model Group were significantly higher than those in the Control Group (5.07 % ± 0.63 % vs. 1.31 % ± 0.10 %, p=0.000; 11.09 % ± 1.20 % vs. 5.73 % ± 0.56 %, p=0.000); Central anti-inflammation by minocycline significantly increased the expression percent of RT1.D in the Sepsis(87.41 % ± 3.35 % vs. 64.18 % ± 2.05 %, p=0.000) and decreased the percents of TH17 and Treg lymphocyte percents in sepsis (1.79 % ± 0.33 % vs. 5.07 % ± 0.63 %, p=0.001;6.93 % ± 0.28 % vs. 11.09 % ± 1.20 %, p=0.000); whereas, the CAP transection by right vagotomy cancelled the effect of central anti-inflammation in sepsis to some extent (RT1.D expression: 77.03 % ± 4.29 % vs. 87.41 % ± 3.35 %, p=0.015; TH17 lymphocytes percent: 2.69 % ± 0.34 % vs. 1.79 % ± 0.33, p=0.120; Treg lymphocytes percent: 9.09 % ± 0.20 % vs. 6.93 % ± 0.28 %, p=0.030).

Similarly, in the neuroinflammation experiment, the expression percent of RT1.D significantly decreased in the neuroinflammation Model Group than that in the Control Group (63.65 % ± 0.42 % vs. 87.55 % ± 3.09 %, p=0.000), on the contrary, the percents of TH17 and Treg lymphocyte percents in the neuroinflammation Model Group were significantly higher than those in the Control Group (6.10 % ± 0.95 % vs. 2.20 % ± 0.17 %, p=0.000;8.87 % ± 0.23 % vs. 3.50 % ± 0.56 %, p=0.000); central anti-inflammation by minocycline significantly increased the expression percent of RT1.D (81.55 % ± 0.96 % vs. 63.65 % ± 0.42 %, p=0.000) and decreased the percents of TH17 and Treg lymphocyte in the neuroinflammation models (2.90 ± 0.26 % vs. 6.10 % ± 0.95 %, p=0.001; 6.10 % ± 0.20 % vs. 8.87 % ± 0.23 %, p=0.000); whereas, the CAP transection by right vagotomy cancelled the effect of central anti-inflammation in sepsis (RT1.D expression: 76.21 % ± 2.86 % vs. 81.55 % ± 0.96 %, p=0.017; TH17 lymphocytes percent: 4.40 % ± 0.46 % vs. 2.90 ± 0.26 %, p=0.010; Treg lymphocytes percent: 7.27 % ± 0.35 % vs. 6.10 % ± 0.20 %, p=0.004).

The serum concentration of TNF-a (ng/L), IL-10(ng/L), INF-γ (ng/L) in the sepsis Model Group were significantly higher than those in the Control Group (32.84 ± 1.39 vs. 9.51 ± 1.50, p=0.000; 19.01 ± 1.21 vs. 5.67 ± 1.07, p=0.000; 12.18 ± 0.24 vs. 7.28 ± 0.21, p=0.000); They decreased significantly in the Anti-inflammatory Group compared to the Model Group (16.46 ± 1.55 vs. 32.84 ± 1.39, p=0.000; 9.44 ± 1.03 vs. 19.01 ± 1.21, p=0.000; 11.00 ± 0.40 vs. 12.18 ± 0.24, p=0.000), whereas, in the CAP Transection Group, they had marvelous rebounds compared to the Anti-inflammatory Group (27.36 ± 1.48 vs. 16.46 ± 1.55, p=0.000; 14.08 ± 0.78 vs. 9.44 ± 1.03; p=0.001; 8.82 ± 0.58 vs. 11.00 ± 0.40, p=0.000).

Similarly, the concentrations of TNF-a (ng/L), IL-10(ng/L), INF-γ (ng/L) in the neuroinflammation Model Group were significantly higher than those in the Control Group (67.29 ± 3.49 vs. 14.16 ± 2.00, p=0.000; 11.89 ± 0.54 vs. 6.31 ± 0.49, p=0.000; 14.38 ± 0.67 vs. 9.62 ± 0.56, p=0.000); They significantly decreased in the Anti-inflammation Group compared to the Model Group (26.24 ± 4.43 vs. 67.29 ± 3.49, p=0.000; 8.00 ± 0.53 vs. 11.89 ± 0.54, p=0.001; 10.70 ± 0.53 vs. 14.38 ± 0.67, p=0.001); the concentrations of TNF-a (ng/L), IL-10(ng/L) in the CAP Transection Group increased significantly compared to the Central Anti-inflammatory Group (47.20 ± 4.78 vs. 26.24 ± 4.43, p=0.001; 10.35 ± 0.69 vs. 8.00 ± 0.53, p=0.006), whereas, the concentrations of INF-γ (ng/L) had the tendency to decrease in the CAP Transection Group when compared that in the Anti-inflammatory Group (11.96 ± 0.55 vs. 8.00 ± 0.53, p=0.174).

## Discussion

This study demonstrated that sepsis-induced neuroinflammation in the medullary visceral zone (MVZ) activates glial cells, impairs synaptic plasticity, and inhibits the cholinergic anti-inflammatory pathway (CAP), thereby aggravating peripheral cytokine storm and immune disturbance. Early central anti-inflammatory intervention with minocycline alleviated glial activation, restored synaptic structure-related protein expression, improved CAP regulatory function, and reduced systemic inflammation. These beneficial effects were partially reversed by CAP transection, confirming that the CAP pathway plays a critical role in mediating central–peripheral inflammatory crosstalk during sepsis.

### Glial activation in the MVZ contributes to sepsis-induced neuroinflammation

Glial cells are decisive in the initiation and maintenance of neuroinflammation [3]. Upon activation by pro-inflammatory cytokines, microglia undergo morphological changes, transitioning from dendritically quiescent M2 microglia to amebic pro-inflammatory M1 microglia, and up-regulate the expression of Iba-1 and NO levels [20]. Iba-1 is a marker of activated microglia and macrophages, and CD11b is a marker of M1 microglia [21]. Lipopolysaccharide (LPS) has been shown to interact with toll-like receptor (TLR) 4 on microglia membranes, initiating an immune cascade, with 1 μg/mL LPS capable of activating microglia and inducing acute neuroinflammation [22], 23]. Glial fibrillary acidic protein (GFAP) is a biomarker of astrocyte stimulation, expressed at low levels in healthy human brains [24]. GFAP expression is significantly increased in neurodegeneration, injury, neuroinflammation, Alzheimer’s disease (AD) [25], 26], and other pathological conditions, playing a key role in initiating AD [27]. GFAP is involved in the regulation of neuroinflammation, nerve repair, and other functions [28], 29]. In sepsis experiments, it follows that systemic inflammation can activate microglia and astrocytes, further promoting nerve injury and dysfunction. Minocycline inhibits neuroinflammation by inhibiting the polarization of microglia, inhibiting the synthesis of inducible nitric oxide synthase, and reducing the phosphorylation of p38 mitogen-activated protein kinase (MAPK) [30]. Additionally, minocycline can inhibit the activation of caspase-1 and caspase-3, playing an anti-apoptotic role [31].

In the present study, both the peripheral sepsis model and central MVZ neuroinflammation model showed significantly upregulated expression of GFAP, Iba-1, and CD11b in the MVZ, indicating robust activation of astrocytes and microglia. Minocycline, a selective microglial inhibitor with anti-neuroinflammatory and neuroprotective effects [8], markedly suppressed glial activation in both models. These results are consistent with previous reports that minocycline inhibits microglial polarization, reduces pro-inflammatory mediator synthesis, and exerts anti-inflammatory and anti-apoptotic effects [30], 31].

Notably, CAP transection partially abolished the anti-inflammation effect of minocycline, suggesting that CAP is a critical neuro-immunity path to modulate the systemic inflammation. Taken together, these findings confirm that glial activation in the MVZ is a core pathological event in sepsis-induced neuroinflammation, and that central anti-inflammatory intervention targeting glia may break the vicious cycle between neuroinflammation and systemic inflammation.

### Impaired synaptic plasticity links MVZ neuroinflammation to CAP dysfunction

Synaptic structural integrity is essential for normal neural signal conduction. Previous studies have confirmed that neuroinflammation disrupts synaptic structure and synaptic-related protein expression [10], 32]. A series of proteins function in controlling vesicle movement and neurotransmitter release, among which Munc13 plays a key role in the timely and precise release of neurotransmitters by promoting fusion between the presynaptic membrane and vesicles. It is generally believed that Munc13 forms hexamers on the lipid bilayer of the presynaptic membrane to trap vesicles and control the quantum size of neurotransmitter release [33]. In Munc13-1 knockout neurons, although completely normal synapses could be formed, the neurotransmitter release process was almost completely blocked [34]. The downregulated expression of Munc13-1 indicates an obstacle to efferent nervous information.

PSD-95 lies in the postsynaptic membrane, an important component of the receptor scaffold protein, facilitating the formation and stabilization of the receptor complex [35]. In fact, PSD-95 dynamically changes in quantity and function to control the number of active postsynaptic receptors [36]. The number, size, and density of PSD-95 protein nanoclusters vary according to synapse types, degrees of development, and plasticity, thus adapting to multiple functional states of synapses [37]. Therefore, the dynamic changes of PSD-95 and other components in the postsynaptic active zone are related to the number of presynaptic vesicles and synaptic plasticity [38]. A key feature of excitatory synapses is the sub-synaptic protein nanoclusters (NCs), their precise arrangement across the synaptic cleft, and forming the synaptic nanopillar affect the strength of synaptic transmission. Reduced PSD-95 levels can lead to synaptic defects [39], associated with pathologies such as weight loss [40], depression [41], and dementia [42].

In this study, both sepsis and MVZ neuroinflammation induced significantly decreased expression of Munc13-1, SYN, and PSD-95. Minocycline treatment partially upregulated synaptic plasticity related protein levels, whereas CAP transection offset these improvements.

These results indicate that sepsis-induced MVZ neuroinflammation impairs synaptic plasticity, which may disrupt neuro-immunity signal transmission from MVZ to peripheral immunity organs or cells through CAP. We suggest that synaptic plasticity damage serves as a key intermediate link between glial activation and CAP inhibition during sepsis.

### CAP inhibition mediates systemic inflammation and immune imbalance

The CAP is a key neural pathway that suppresses peripheral inflammatory responses through the vagus nerve [1], 2], 43], 44]. Heart rate variability (HRV) parameters, including SDNN, RMSSD, LF, and HF, can reflect CAP regulatory strength [2], 44]. In this study, both models showed significantly reduced HRV indices, indicating CAP inhibition. Minocycline restored these parameters, and vagotomy abolished this effect, verifying the CAP-dependent mechanism.

At the peripheral level, CAP inhibition was accompanied by elevated serum TNF-α, IL-10, and IFN-γ levels. TNF-α is a pivotal pro-inflammatory cytokine that triggers inflammatory cascades [45], while IFN-γ promotes macrophage polarization toward a pro-inflammatory phenotype [46], 47]. IL-10 is an anti-inflammatory cytokine that increases during excessive inflammation to limit immune overactivation [48].

In terms of immune profiles, both models showed decreased RT1B (MHC II) expression on monocytes, which is associated with immunosuppression and poor prognosis in sepsis [49], 50]. Meanwhile, the proportions of Th17 and Treg lymphocytes were increased. Th17 cells promote inflammatory responses, whereas Treg cells maintain immune homeostasis [51], [52], [53]. Minocycline reversed these immune changes, and these effects were partially reversed by CAP transection. These results demonstrate that MVZ neuroinflammation drives systemic inflammation and immune disturbance mainly by inhibiting CAP.

### Rationale for similar outcomes between sepsis and MVZ neuroinflammation models

A notable finding is that peripheral sepsis and central MVZ neuroinflammation triggered highly consistent changes in glial activation, synaptic plasticity, CAP function, and peripheral inflammation. This similarity arises because both models converge on MVZ glial activation and synaptic plasticity damage, which further disable CAP modulation of systemic immunity.

Peripheral LPS induces systemic inflammation that activates MVZ glia indirectly across the blood–brain barrier, whereas central LPS activates MVZ glia directly. In both scenarios, CAP dysfunction is the key event leading to peripheral cytokine storm and immune dysregulation. This parallel outcome strongly supports that MVZ neuroinflammation is a dominant mechanism underlying systemic inflammation in sepsis.

### Clinical implications

The present findings provide potential translational strategies for sepsis treatment. First, early central anti-inflammatory intervention targeting MVZ glial activation may represent a novel approach to control sepsis progression. Second, HRV indices can serve as a non-invasive biomarker to monitor CAP function and MVZ inflammation severity. Third, protecting synaptic plasticity in the MVZ may help preserve vagal anti-inflammatory output and limit inflammatory escalation.

### Limitations

Several limitations should be acknowledged.

1. No naive control group without intracerebroventricular injection was included. Although the ACSF control showed minimal glial activation, a non-injected control would further exclude surgical interference.
2. Synaptic plasticity was not directly measured by electrophysiological methods such as LTP/LTD; only synaptic structure and protein expression were evaluated.
3. All animals were male rats; sex differences in sepsis-induced neuroinflammation remain unclear.
4. The model reflects the acute inflammatory stage of sepsis and does not address the late immunosuppressive phase.
5. This is an animal study; clinical studies are needed to verify translatability to human sepsis.

## Conclusions

This study demonstrates that sepsis induces neuroinflammation in the MVZ, which impairs synaptic plasticity by activating glial cells, leading to functional inhibition of the CAP, and promoting systemic immune activation and inflammatory storm. The neuroinflammation of the MVZ likely dominates the inflammatory and immune disorders in sepsis. Central anti-inflammatory treatment may become a critical intervention strategy for inflammatory and immune disorders in sepsis.
